# Sexual health and COVID-19: protocol for a scoping review

**DOI:** 10.1186/s13643-021-01591-y

**Published:** 2021-01-23

**Authors:** Navin Kumar, Kamila Janmohamed, Kate Nyhan, Laura Forastiere, Wei-Hong Zhang, Anna Kågesten, Maximiliane Uhlich, Sarah M. Van de Velde, Joel M. Francis, Jennifer T. Erausquin, Elin C. Larsson, Denton Callander, John Scott, Victor Minichiello, Joseph D. Tucker

**Affiliations:** 1 Human Nature Lab, Department of Sociology, Yale University, New Haven, CT USA; 2 Yale College, New Haven, CT USA; 3 Harvey Cushing/John Hay Whitney Medical Library, Yale University, 333 Cedar Street, New Haven, CT 06520-8014 USA; 4 Department of Environmental Health Sciences, Yale School of Public Health, New Haven, CT USA; 5 Department of Biostatistics, Yale School of Public Health, New Haven, CT USA; 6 International Centre for Reproductive Health, Department of Public Health and Primary Care, Ghent University, Ghent, Belgium; 7 School of Public Health, Universit́e Libre de Bruxelles, Brussels, Belgium; 8 Department of Global Public Health, Karolinska Institutet, Stockholm, Sweden; 9 Department of Psychology, Universit́e de Fribourg, Fribourg, Switzerland; 10 Department of Sociology, Centre for Population, Family and Health, University of Antwerp, Sint-Jacobstraat 2-4, 2000 Antwerp, Belgium; 11 Department of Family Medicine and Primary Care, School of Clinical Medicine, Faculty of Health Sciences, University of the Witwatersrand, Johannesburg, South Africa; 12 Department of Public Health Education, The University of North Carolina at Greensboro, Greensboro, NC USA; 13 Department of Women’s and Children’s Health, Karolinska Institutet, Stockholm, Sweden; 14 Department of Epidemiology, Mailman School of Public Health, Columbia University, New York, NY USA; 15 Queensland University of Technology, Brisbane, Queensland Australia; 16 Faculty of Medicine and Health, University of New England, Armidale, New South Wales Australia; 17 School of Social Justice, Queensland University of Technology, Brisbane, Queensland Australia; 18 University of North Carolina at Chapel Hill, Project-China, No. 2 Lujing Road, Guangzhou, 510095 China; 19 School of Medicine, University of North Carolina at Chapel Hill, Chapel Hill, NC USA; 20 Faculty of Infectious and Tropical Diseases, London School of Hygiene and Tropical Medicine, London, UK

**Keywords:** COVID-19, Sexual health, Sexual minority, LGBT, Women

## Abstract

**Background:**

Global responses to the COVID-19 pandemic have exposed and exacerbated existing socioeconomic and health inequities that disproportionately affect the sexual health and well-being of many populations, including people of color, ethnic minority groups, women, and sexual and gender minority populations. Although there have been several reviews published on COVID-19 and health disparities across various populations, none has focused on sexual health. We plan to conduct a scoping review that seeks to fill several of the gaps in the current knowledge of sexual health in the COVID-19 era.

**Methods:**

A scoping review focusing on sexual health and COVID-19 will be conducted. We will search (from January 2020 onwards) CINAHL, Africa-Wide Information, Web of Science Core Collection, Embase, Gender Studies Database, Gender Watch, Global Health, WHO Global Literature on Coronavirus Disease Database, WHO Global Index Medicus, PsycINFO, MEDLINE, and Sociological Abstracts. Grey literature will be identified using Disaster Lit, Google Scholar, governmental websites, and clinical trials registries (e.g., ClinicalTrial.gov, World Health Organization, International Clinical Trials Registry Platform, and International Standard Randomized Controlled Trial Number Registry). Study selection will conform to the Joanna Briggs Institute Reviewers’ Manual 2015 Methodology for JBI Scoping Reviews. Only English language, original studies will be considered for inclusion. Two reviewers will independently screen all citations, full-text articles, and abstract data. A narrative summary of findings will be conducted. Data analysis will involve quantitative (e.g., frequencies) and qualitative (e.g., content and thematic analysis) methods.

**Discussion:**

Original research is urgently needed to mitigate the risks of COVID-19 on sexual health. The planned scoping review will help to address this gap.

**Systematic review registrations:**

Systematic Review Registration: Open Science Framework osf/io/PRX8E

**Supplementary Information:**

The online version contains supplementary material available at 10.1186/s13643-021-01591-y.

## Background

Global responses to the COVID-19 pandemic have exposed and exacerbated existing socioeconomic and health inequities that disproportionately affect the health and well-being of people of color, ethnic minority groups, women, and sexual and gender minority populations [1–3]. Many sub-populations may experience worse sexual health during COVID-19. Sexual health research, broadly defined, is the study of an individuals’ physical, emotional, mental, and social well-being in relation to sexuality; it goes beyond the absence of disease, dysfunction, or infirmity [4]. In this respect, sexual health has psycho-social dimensions, in addition to physical dimensions. Sexual health research includes studies that center on sexual minorities as a population and comprises sexual behavior and access to high-quality sexual health care. For the purposes of the planned review, due to a lack of research and that systematic reviews on these areas are currently being conducted, reproductive health, intimate partner violence, and gender-based violence will not be considered components of sexual health. Guidelines to stay at home, the resulting economic impact on individuals and families, and the need to shift healthcare resources (including money, clinic space, and staff) to the COVID-19 response are likely to affect sexual behavior, sexual health, and access to quality sexual health care. Research suggests that a reduction in economic opportunities may impact sexual healthcare access for women [5]. Similarly, during health crises, sexual health resources may be diverted to the pandemic response, with the potential to increase maternal mortality and limit abortion care and contraception access [6, 7]. Sex workers worldwide may see clients in person, risking infection and perhaps not seeking medical care due to reduced healthcare provision [8]. In some countries, like the USA, the LGBTQIA community is also less likely to have health insurance [9], increasing negative economic impacts if they contract COVID-19. These factors may widen socioeconomic inequity and further reduce access to sexual health services. Key populations experience unique challenges in the wake of the pandemic including delays in seeking treatment due to fear of stigma, discrimination, and involuntary outing of sexual orientation or immigration status through contact tracing and isolation [10].

Several published reviews have focused on COVID-19 and health outcomes across various populations [11, 12]. However, these reviews did not center on sexual health, an area of health and well-being potentially negatively affected by the pandemic. Sexual health is key to overall human health and well-being and to the socioeconomic development of communities and countries [13]. Moreover, people of color, women, and ethnic and sexual minorities likely face greater negative impacts from the pandemic, especially sexual health. The planned scoping review seeks to compile published evidence in the field to identify gaps in the current understanding of sexual health and COVID-19. We will conduct a scoping review rather than use other methods of research synthesis because scoping reviews are appropriate for mapping an area of research [14]; we will not be examining the effect of an intervention on an outcome of interest, and thus, it does not make sense to assess the risk of bias, as per a systematic review; and sexual health research outcomes are likely not sufficiently similar to each other to warrant pooling or formal meta-analysis regarding a specific outcome. The review will include and contrast research detailing sexual health and COVID-19 among individuals of all genders and sexual identities [4]. This review will also include research that has examined a broad range of outcomes and studies related to sexual health and well-being (e.g., testing, risk behaviors, treatment, PrEP use, vaccination, gender-based violence, selling sex).

The planned scoping review will build on existing reviews around COVID-19 and a range of other populations and health outcomes. Our review will chart the change in sexual health research, such as shifts in topics of focus and research technique (e.g., qualitative versus quantitative), to determine if some research areas are tied to changes in pandemic progression. Key to the development of interventions that improve sexual health amid COVID-19 is a comprehensive understanding of the current status of evidence around sexual health during the COVID-19 era. The planned scoping review seeks to understand the gaps in the current knowledge base on sexual health and COVID-19, especially in marginalized group such as sexual minorities and people living with HIV. The planned scoping review seeks to provide this evidence by contributing an evaluation of available literature about sexual health in relation to COVID-19, with the goal of identifying gaps in research.

## Methods/design

The review protocol has been registered within the Open Science Framework database (osf/io/PRX8E) and is being reported in accordance with the reporting guidance provided in the Preferred Reporting Items for Systematic Reviews and Meta-Analyses Protocols (PRISMA-P) statement [15] (see checklist in Additional file 1). The proposed scoping review will be reported in accordance with the reporting guidance provided in the Preferred Reporting Items for Systematic Reviews and Meta-analyses (PRISMA) extension for Scoping Reviews (PRISMA-ScR) [16]. Research objectives, inclusion criteria, and methodological techniques will be determined before study commencement using the Joanna Briggs Institute Reviewers’ Manual 2015 Methodology for JBI Scoping Reviews [17]. This process will adhere to the indicated framework: (1) identifying research question, (2) developing comprehensive search strategy, (3) identifying relevant studies, (4) selecting studies, (5) charting data, and (6) collating, summarizing, and reporting results. The study team will develop a search strategy as recommended by the 2015 Methodology for JBI Scoping Reviews.

This scoping review will be conducted by 13 individuals: 12 researchers from several universities worldwide, from a range of disciplines (e.g., medicine, sociology, demography, public health, criminology, economics, psychology, epidemiology), and an informationist from the Harvey Cushing/John Hay Whitney Medical Library at Yale University. The objective of the scoping review is to develop a better understanding of the current research landscape around sexual health and COVID-19 by investigating the existing studies and gaps in the research. The broad research questions are “what has been reported on sexual health in the COVID-19 era?” and “what are the gaps in the current knowledge base on sexual health and COVID-19 across diverse populations, including marginalized groups?” The search strategy will be performed in line with techniques that enhance methodological transparency and improve the reproducibility of the results and evidence synthesis.

### Information sources and search strategy

The primary source of literature will be a structured search of electronic databases (from January 2020 onwards): MEDLINE, EMBASE, CINAHL, PsycINFO, Web of Science Core Collection, Africa-Wide Information, Gender Studies Database, Gender Watch, Global Health, WHO Global Literature on Coronavirus Disease Database, WHO Global Index Medicus, and Sociological Abstracts. The secondary source of potentially relevant material will be a search of preprint servers (e.g., medRxiv.org, PsyArXiv.org), Disaster Lit, Google Scholar (e.g., the first five pages will be searched), governmental websites, and clinical trials registries (e.g., ClinicalTrial.gov, World Health Organization International Clinical Trials Registry Platform, and International Standard Randomized Controlled Trial Number Registry). The references of included documents will be hand-searched to identify any additional evidence sources. The search strategy will be designed by a research librarian and peer-reviewed by using the Peer Review of Electronic Search Strategies (PRESS) checklist [18]. A draft search strategy for MEDLINE is provided in Additional file 2.

We will use search terms similar to our main search to find articles for inclusion.

The same keywords for the main search will be used to search grey literature each time. All grey literature will be compiled in a folder and reviewed similarly to articles obtained from our database searches. EndNote, a bibliographic software, will be used to store, organize, and manage all references [19].

### Eligibility criteria

We will include all studies with all study designs involving COVID-19 and sexual health. Only English language studies will be considered for inclusion. Past work indicated that excluding non-English language records from a review seemed to have a minimal effect on the results [20, 21].

#### Inclusion criteria

Published research (peer-reviewed and grey literature where primary data was collected such as reports, research letters, and briefs) investigating sexual health and COVID-19 in all populations, settings, and study designs, e.g., studies with small samples, quantitative and qualitative studies, will be eligible for inclusion. We will include studies focusing on sex workers, LBTQIA persons, and persons at risk for HIV, even if these studies do not examine sexual health specifically. Primary outcomes will include how the COVID-19 pandemic affects sexual health, both effects of the lockdown and the biological impact of the virus on sexual health, and how the COVID-19 pandemic affects sexual minorities. Primary outcomes will not include reproductive health, intimate partner violence, and gender-based violence alone.

There will be no restrictions on age, region, or gender.

Studies reported only as conference abstracts will be included, only if we do not have access to the full paper. Conference abstracts are often left out of systematic reviews as they may not contain adequate information to conduct quality assessment or a meta-analysis. Here, we will include conference abstracts as they are often published earlier than full manuscripts [22], which is key to a thorough scoping review on an ongoing phenomenon.

#### Exclusion criteria

Commentaries, correspondences, case reports, case series, editorials, and opinion pieces will be excluded. Case reports and case series often contain relatively limited evidence [23].

Governmental or other agency guidelines will be excluded.

Reviews such as systematic reviews and scoping reviews will be excluded, but we will review the references in these for inclusion, if applicable.

### Screening and selection procedure

All reports identified from the searches will be screened by two reviewers independently. First, titles and abstracts of articles returned from initial searches will be screened based on the eligibility criteria outlined above. Second, full texts will be examined in detail and screened for eligibility. Third, references of all considered articles will be hand-searched to identify any relevant report missed in the search strategy. Any disagreements will be resolved by discussion, or if necessary, with a third reviewer. A flow chart showing details of studies included and excluded at each stage of the study selection process will be provided. We will contact the authors where necessary if the abstracts do not provide sufficient information [22]. Covidence will be used to manage the title/abstract and full-text screening phases [24].

### Data extraction

Reviewers will undergo practice exercises till they have a high level of agreement (*>* 0.8 kappa) and then independently extract data from studies. Reviewers will abstract the data using a pretested data extraction template. We will use a standardized coding protocol to collect information such as title of study, authors, date published, author affiliation as a measure to ascertain the discipline focus of the study and collaborating institutions, study setting, study design, description of methodology, description of the study sample, definition or type of sexual health studied (if any), measurements and scales used, main findings, funder information, journal title, and submission variant (research letter, short report, original article, etc.). Even though a formal risk of bias is not planned for this scoping review, we will note which studies are pre-prints, and thus, have not been formally peer-reviewed.

### Data synthesis

Outcomes and other information collected regarding selected studies will be synthesized using quantitative (e.g., frequencies) and qualitative (e.g., content and thematic analysis) methods, with a narrative summary of findings conducted. The synthesis will be presented in tables, summary data in graphs, and individual data for each study in tables. The broad goal of the synthesis is to identify the gaps in research and present recommendations for future research agendas.

## Discussion

The strength of the planned scoping review is the use of a transparent and reproducible procedure for a scoping literature review. We state the data sources, search strategy, and data extraction [25]. Through publishing this research protocol, we strengthen the clarity of the search strategy.

There have been few studies which compile available evidence from various settings in relation to sexual health and COVID-19. Our review will provide an overview of these studies, synthesizing evidence. There is much anecdotal work around sexual health and COVID-19, with few published studies. The planned review will highlight the areas of research focus and gaps which require more attention. Moreover, the COVID-19 context is quickly changing [26] likely affecting sexual health in a rapidly shifting fashion. Results will thus provide high-level information to inform, support, and customize design of interventions to mitigate reduced sexual health outcomes in this setting. As researchers attempt to minimize the harms from COVID-19, especially for marginalized populations (e.g., people of color, ethnic minority groups, women, and sexual and gender minority populations), they need to be aware of scientific evidence to develop interventions to achieve their aim. The planned scoping review seeks to provide this evidence by contributing an evaluation of what is currently known about sexual health in relation to COVID-19, with the goal of identifying gaps in research and presenting recommendations for future research foci.

Any amendments to this protocol will be documented in the final published scoping review with reference to saved searches and analysis.

Results of the review will be disseminated in a peer-reviewed journal and likely in other media such as conferences, seminars, and symposia. The protocol and final review article will be made open access upon publication. As per PRISMA-ScR guidelines, we will present results in a user-friendly format [27].

### Limitations

Our planned review should be read in line with some limitations. Although we plan to search several databases and gray literature sources, we may miss some studies. Not all authors we reach out to may respond, and we may thus miss some unpublished work. We may not be able to make policy recommendations due to the lack of quality appraisal of studies [28].

## Supplementary Information

**Additional file 1:.** Preferred Reporting Items for Systematic reviews and Meta-Analyses extension for Scoping Reviews (PRISMA-ScR) Checklist.

**Additional file 2:.** Search strategy.

## Data Availability

The datasets used and analyzed will be made available from the authors upon reasonable request.
