# Supplementary material for: Sexual health and COVID-19: protocol for a scoping review
Source: Syst Rev. 2021 Jan 23;10:37. doi: 10.1186/s13643-021-01591-y (PMC7825389; doi:10.1186/s13643-021-01591-y)
Supplement: Supplementary file 2 — Additional file 2:. Search strategy. [file 13643_2021_1591_MOESM2_ESM.pdf]

MEDLINE DRAFT SEARCH STRATEGY

Search strategy

1	exp Coronavirus/
2	exp Coronavirus Infections/
3	(coronavirus* or corona virus* or OC43 or NL63 or 229E or HKU1 or HCoV* or ncov* or covid* or sars-cov* or sarscov* or Sars-coronavirus* or Severe Acute Respiratory Syndrome Coronavirus*).mp.
4	(or/1-3) and ((20191* or 202*).dp. or 20190101:20301231.(ep).) [this set is the sensitive/broad part of the search]
5	4 not (SARS or SARS-CoV or MERS or MERS-CoV or Middle East respiratory syndrome or camel* or dromedar* or equine or coronary or coronal or covidence* or covidien or influenza virus or HIV or bovine or calves or TGEV or feline or porcine or BCoV or PED or PEDV or PDCoV or FIPV or FCoV or SADS-CoV or canine or CCov or zoonotic or avian influenza or H1N1 or H5N1 or H5N6 or IBV or murine corona*).mp. [line 5 removes noise in the search results]
6	((pneumonia or covid* or coronavirus* or corona virus* or ncov* or 2019-ncov or sars*).mp. or exp pneumonia/) and Wuhan.mp.
7	(2019-ncov or ncov19 or ncov-19 or 2019-novel CoV or sars-cov2 or sars-cov-2 or sarscov2 or sarscov-2 or Sars-coronavirus2 or Sars-coronavirus-2 or SARS-like coronavirus* or coronavirus-19 or covid19 or covid-19 or covid 2019 or ((novel or new or nouveau) adj2 (CoV on nCoV or covid or coronavirus* or corona virus or Pandemi*2)) or ((covid or covid19 or covid-19) and pandemic*2) or (coronavirus* and pneumonia)).mp.
8	COVID-19.rx,px,ox. or severe acute respiratory syndrome coronavirus 2.os.
9	("32240632" or "32236488" or "32268021" or "32267941" or "32169616" or "32267649" or "32267499" or "32267344" or "32248853" or "32246156" or "32243118" or "32240583" or "32237674" or "32234725" or "32173381" or "32227595" or "32185863" or "32221979" or "32213260" or "32205350" or "32202721" or "32197097" or "32196032" or "32188729" or "32176889" or "32088947" or "32277065" or "32273472" or "32273444" or "32145185" or "31917786" or "32267384" or "32265186" or "32253187" or "32265567" or "32231286" or "32105468" or "32179788" or "32152361" or "32152148" or "32140676" or "32053580" or "32029604" or "32127714" or "32047315" or "32020111" or "32267950" or "32249952" or "32172715").ui. [Articles not captured by this search when created in April 2020, pending further indexing by NLM]
10	or/6-9 [Lines 6 to 9 are specific to Covid-19]
11	5 or 10
12	11 and 20191201:20301231.(dt).
13	(covid* or ncov* or 2019-novel CoV or SARS-CoV2 or SARS-CoV-2 or SARSCoV2 or SARSCov-2 or "severe acute respiratory syndrome coronavirus 2").mp.

14 (coronavirus* or corona virus*).mp. and 2020*.dp.
15 ((novel or new or ”2019” or ”19” or pandemic or crisis or outbreak or Wuhan or China) adj3 (coronavirus* or corona virus*)).mp.
16 Covid-19.rx.
17 coronavirus infections/ and 2020*.dp.
18 Pneumonia, Viral/ and 2020*.dp.
19 or/13-18
20 12 or 19
21 [from the CADTH hedge]
22 (coronavirus/ or betacoronavirus/ or coronavirus infections/) and (disease outbreaks/ or epidemics/ or pandemics/)
23 (nCoV* or 2019nCoV or 19nCoV or COVID19* or COVID or SARS-COV-2 or SARSCOV-2 or SARSCOV2 or Severe Acute Respiratory Syndrome Coronavirus 2 or Severe Acute Respiratory Syndrome Corona Virus 2).ti,ab,kf,nm,ot,ox,rx,px.
24 ((new or novel or ”19” or ”2019” or Wuhan or Hubei or China or Chinese) adj3 (coronavirus* or corona virus* or betacoronavirus* or CoV or HCoV)).ti,ab,kf,ot.
25 ((coronavirus* or corona virus* or betacoronavirus*) adj3 (pandemic* or epidemic* or outbreak* or crisis)).ti,ab,kf,ot.
26 ((Wuhan or Hubei) adj5 pneumonia).ti,ab,kf,ot.
27 or/22-26
28 limit 27 to yr=”2019 -Current”
29 [let’s compare]
30 20 [homegrown]
31 27 [CADTH]
32 [additional pandemic terms]
33 (pandemic* or quarantine* or social* distan* or lockdown*).mp.
34 exp disease outbreaks/
35 20 or 27 or 33 or 34
36 limit 35 to yr=”2019 -Current” [confirm this]
37 [sexual health]
38 sexual health/
39 exp sexually transmitted diseases/
40 exp sexual behavior/
41 sexual health.mp.
42 ((sexually transmitted or venereal) adj1 (disease* or infection*)).mp.
43 (std or stds or sti or stis).mp.
44 (gonorrhea or chlamydia or syphilis or herpes).mp.
45 (HIV or human immunodeficiency virus).mp.
46 (AIDS or acquired immunodeficiency syndrome).mp.
47 sexual behavio*.mp.
48 (safe* sex or courtship* or masturbat* or abstinen* or unsafe sex).mp.
49 sexualit*.mp.
50 (sex adj1 (work* or industr*)).mp.
51 prostitut*.mp.

52 (sex and client*).mp.
53 escort*.mp.
54 (brothel* or strip club*).mp.
55 exp sex offenses/
56 domestic violence/ or spouse abuse/
57 ((spouse* or spousal or wife or wives or husband* or partner* or boyfriend* or girlfriend* or domestic) adj2 (abus* or violen*)).mp.
58 (dv or ipv).mp.
59 coercive control.mp.
60 (rape* or rapist*).mp.
61 (sex* adj1 (offen* or violen* or abus* or exploit*)).mp.
62 traffick*.mp.
63 (dating or romantic* or intimate or sexual* or sext* or seksbudd* or sex budd*).mp.
64 sex* partner*.mp.
65 sexual partners/
66 Pre-Exposure Prophylaxis/
67 Post-Exposure Prophylaxis/
68 (exposure prophylaxis or PrEP or PEP or truvada).mp.
69 Emtricitabine, Tenofovir Disoproxil Fumarate Drug Combination/
71 (gender* adj2 nonconform*).mp.
72 (gender* adj2 non-conform*).mp.
73 (trans adj (female* or male* or man or men or women or woman or boy* or girl*)).mp.
74 (trans adj (population* or patient* or participant* or subject* or adolescent* or teen* or child* or individual* or people or person* or youth*)).mp.
75 agender*.mp.
76 bicurious.mp.
77 bigender*.mp.
78 bisexual*.mp.
79 cross sex.mp.
80 crossgender.mp.
81 DSD.mp.
82 gay.mp.
83 gays.mp.
84 gender change.mp.
85 gender crossing.mp.
86 gender dysphori*.mp.
87 gender fluid*.mp.
88 gender identit*.mp.
89 gender incongruen*.mp.
90 gender minorit*.mp.
91 gender neutral.mp.
92 gender queer.mp.
93 gender transition*.mp.
94 gender varian*.mp.

95 genderless.mp.
96 genderqueer*.mp.
97 GLB.mp.
98 GLBQ.mp.
99 GLBs.mp.
100 GLBT.mp.
101 GLBTQ.mp.
102 heteroflexible.mp.
103 homosexual*.mp.
104 intersex*.mp.
105 lesbian*.mp.
106 lesbigay*.mp.
107 LGB.mp.
108 LGBQ.mp.
109 LGBS.mp.
110 LGBT*.mp.
111 men who have sex with men.mp.
112 mostly-heterosexual.mp.
113 MSM.mp.
114 MSMW.mp.
115 nonbinary.mp.
116 non-binary.mp.
117 nonheterosexual*.mp.
118 non-heterosexual*.mp.
119 queer.mp.
120 queers.mp.
121 same gender loving.mp.
122 same sex couple*.mp.
123 same sex relations*.mp.
124 same-sex attract*.mp.
125 sexual identit*.mp.
126 sexual minorit*.mp.
127 sexual orientation*.mp.
128 sexual preference*.mp.
129 SGM.mp.
130 third gender*.mp.
131 transboy*.mp.
132 transex*.mp.
133 transfemale*.mp.
134 transfeminine.mp.
135 transgender*.mp.
136 transgirl*.mp.
137 transmn.mp.
138 transmasculine.mp.
139 transmale*.mp.
140 transsex*.mp.

141 trans-sex*.mp.
142 trans-spectrum.mp.
143 transwomn.mp.
144 two-spirit*.mp.
145 women loving women.mp.
146 women who have sex with women.mp.
147 WSW.mp.
148 WSWM.mp.
149 exp ”sexual and gender minorities”/
150 bisexuality/
151 transsexualism/
152 exp homosexuality/
153 gender identity/
154 health services for transgender persons/
155 gender dysphoria/
156 exp ”Disorders of Sex Development”/
158 gender affirming.mp.
159 or/37-157
160 [summation]
161 36 and 159
162 limit 161 to yr=”2020”
163 limit 162 to english language
